# Vaccine Development for Severe Fever with Thrombocytopenia Syndrome

**DOI:** 10.3390/v13040627

**Published:** 2021-04-06

**Authors:** Tomoki Yoshikawa

**Affiliations:** Department of Virology I, National Institute of Infectious Diseases, Musashimurayama-shi, Tokyo 208-8501, Japan; ytomoki@nih.go.jp

**Keywords:** severe fever with thrombocytopenia syndrome, vaccine, animal model

## Abstract

Severe fever with thrombocytopenia syndrome (SFTS), which is caused by SFTS virus (SFTSV), is a tick-borne emerging zoonosis with a high case-fatality rate. At present, there is no approved SFTS vaccine, although the development of a vaccine would be one of the best strategies for preventing SFTS. This article focused on studies aimed at establishing small animal models of SFTS that are indispensable for evaluating vaccine candidates, developing these vaccine candidates, and establishing more practical animal models for evaluation. Innate immune-deficient mouse models, a hamster model, an immunocompetent ferret model and a cat model have been developed for SFTS. Several vaccine candidates for SFTS have been developed, and their efficacy has been confirmed using these animal models. The candidates consist of live-attenuated virus-based, viral vector-based, or DNA-based vaccines. SFTS vaccines are expected to be used for humans and companion dogs and cats. Hence for practical use, the vaccine candidates should be evaluated for efficacy using not only nonhuman primates but also dogs and cats. There is no practical nonhuman primate model of SFTS; however, the cat model is available to evaluate the efficacy of these candidate SFTS vaccines on domesticated animals.

## 1. Introduction

Severe fever with thrombocytopenia syndrome (SFTS) is an emerging viral hemorrhagic fever with a high case-fatality rate (approximately 5% to >40%) [1,2,3,4,5,6]. The clinical symptoms generally include fever, malaise, myalgia, nausea, vomiting, and diarrhea. The laboratory findings include leukocytopenia and thrombocytopenia, as well as elevated serum levels of hepatic enzymes [1,7,8,9,10,11]. Age is a critical risk factor, and morbidity and mortality drastically increase in patients of >50 years of age [4,12]. The disease is caused by SFTS virus (SFTSV), a novel tick-borne virus. SFTSV has been assigned to species *Dabie bandavirus*, which is a species in genus *Bandavirus* in the bunyaviral family *Phenuiviridae* [13]. The viral genome consists of three negative-stranded RNA segments and encodes four genes: RNA-dependent RNA polymerase (RdRp), envelope glycoprotein precursor (GPC), nucleoprotein (N), and nonstructural protein (NSs). Indigenous SFTS has been reported in China, Japan, South Korea, Vietnam, and Taiwan [1,2,3,14,15,16,17,18].

SFTSV is primarily transmitted to animals and humans by ticks. Human-to-human transmission rarely occurs through contact with infected blood, saliva, and possibly aerosols [19,20]. Moreover, domesticated animals, such as companion dogs and cats, should be considered a source of animal-to-human transmission, since viral RNA, specific antibodies, and even virus isolation from sera have reported in various wild and domesticated animals, including dogs and cats [10,21,22,23,24,25]. Although the clinical manifestations of SFTS in domesticated animals are still uncertain, the manifestations observed in cats appear similar to those in humans, and the fatality rate of cats is higher than that of humans [26]. Three cases of cat-to-human transmission of SFTS have been reported [27,28,29]. It is a serious matter that two of the three cases of cat-to-human transmission of SFTS involved veterinarians who had cared for cats infected with SFTSV. These cases indicate that veterinarians who manage infected animals are at a higher risk of SFTSV infection. Moreover, a case report suggests that dogs may infrequently develop SFTS [30]. A dog-related SFTS case has also been reported [31]. However, the patient was likely to have been infected with SFTSV from blood splash after removing or bursting ticks from the dog with their bare hands. Thus, in addition to humans, SFTS vaccine targets include companion dogs and cats. The development of a vaccine would be one of the most effective ways to prevent SFTS. The vaccine should be highly effective at preventing infection, the onset of illness or severe illness, and if feasible, it should be provided at an affordable price.

## 2. Animal Models of SFTS

### 2.1. Mouse Models

Although C57Bl/6, BALB/c, and CD-1 mice are not resistant to SFTSV infection by intravenous, intramuscular, intraperitoneal, or intracerebral routes, they do not develop severe disease. However, they develop hallmark symptoms of thrombocytopenia and leukocytopenia with transient viral replication in the spleen [32,33]. In contrast, two mouse strains on the C57BL/6 background are highly susceptible to SFTSV infection [33,34,35,36]. The mouse strains are deficient of the type I interferon receptor-deficient (*Ifnar^−^*^/*−*^) or signal transducer and activator of transcription 2-deficient (*Stat2^−^*^/*−*^) genes, which are essential for the innate immune signaling pathway. These strains develop severe body weight loss, leukocytopenia, and thrombocytopenia with efficient viral replication and die within one week.

### 2.2. Hamster Model

The Golden Syrian hamster (hamster), the most common laboratory hamster strain, is resistant to SFTSV infection via intravenous, intramuscular, intraperitoneal, or intracerebral routes [32,33]. In contrast, the *Stat2^−^*^/*−*^ hamster is highly susceptible to SFTSV challenge with as few as ten plaque-forming units (PFU) via the subcutaneous route, with infected animals succumbing within one week [37]. The hamsters developed mild bodyweight loss depending on the challenge dose and thrombocytopenia with efficient viral replication, but did not develop leukocytopenia, although neutrophil inversion to lymphocyte numbers is indicative of a robust inflammatory response.

### 2.3. Ferret Model

Thus far, the ferret is the only immunocompetent animal that is susceptible to SFTSV infection [38]. Similar to humans, the susceptibility to SFTSV is age-dependent. Following an SFTSV challenge, young adult ferrets of <2 years of age did not develop symptoms and survived. In contrast, when aged ferrets of >4 years of age were challenged with 10^7.6^ 50% tissue culture infectious dose (TCID_50_) of SFTSV via the intramuscular route, they developed severe thrombocytopenia, leukocytopenia, high fever and succumbed by day eight post-infection. Moreover, the viral load in the organs and serum of the aged ferrets was significantly higher in comparison to young adult ferrets.

### 2.4. Cat Model

Cats developed similar or more severe SFTS than human SFTS patients [39]. Two groups of cats, a 0.5-year-old group and a 2-year-old group, were intravenously infected with 10^7^ TCID_50_/mL of SFTSV. The mortality rate was 67% (four out of six), whereas no relationship was noted between the age and disease severity. The cats developed leukocytopenia, thrombocytopenia, and body temperature elevation. In the cats with a fatal outcome, the viral RNA copies in blood began to increase on day 1, and peaked from days 3–7. RNA was also detected in eye swabs, saliva, and rectum swabs; the RNA levels in these samples peaked on day 7.

### 2.5. Nonhuman Primate Models

Nonhuman primate models of SFTS have not been established. Jin et al. reported that a rhesus macaque developed SFTS, and that the symptoms resembled mild SFTS in humans [40]. Healthy adult female rhesus macaques (age: 4–5 years) were challenged intramuscularly with 1 × 10^7^ TCID_50_ of SFTSV. The macaques showed a mild body temperature increase, leukocytopenia, and thrombocytopenia, but did not develop severe symptoms or die. The viral RNA copies in blood began to increase on day 1, peaked during days 3–5 post-infection, and were undetectable on day 7 post-infection. The viral RNA copies or infectious viral titers during peak viremia ranged from 10^4^ to 10^5^ copies/mL or 10^1.6^ to 10^4.1^ TCID_50_/mL. This range of peak viral RNA copies in blood was similar to that reported in SFTS patients with nonfatal outcomes [41]. These results indicated that SFTSV could productively infect rhesus macaques. Matsuno et al. reported that cynomolgus macaques did not develop SFTS [42]. The macaques were subcutaneously challenged with 10^6^ TCID_50_ of SFTSV or Heartland virus. None of the SFTSV- or HRTV-infected macaques exhibited visible clinical signs, with the exception of one animal infected with SFTSV which showed a temporarily decreased platelet count. Moreover, the blood viral load was below the limit of detection for 14 days, and no macroscopic lesions were found on day 14 post-infection.

## 3. SFTS Vaccine Candidates

### 3.1. Live-Attenuated Virus Vaccines

Historically, live-attenuated viruses are among the most successful vaccine platforms, including the yellow fever 17D vaccine [43] and oral polio vaccine Sabin strains [44]. A classical method for generating the virus involves passaging in cell culture until the virus acquires an attenuated phenotype. Currently, the attenuation is fulfilled by a reverse genetics approach based on knowledge gained from molecular virology. The possible application of live-attenuated viruses as a vaccine candidate for SFTS was studied by Yu et al. [45]. Two recombinant SFTSVs, named rHB2912aaNSs and rHB29NSsP102A, were generated via a reverse genetics approach from SFTSV strain HB29. In both recombinant viruses, the protein-coding region of NSs, which acts as an antagonist to evade innate immunity (e.g., interferon-related signaling), is modified. rHB2912aaNSs is an internal NSs deletion mutant (delta 2–282), which leaves the first methionine and last 11 amino acids [46]. rHB29NSsP102A possesses a proline to alanine amino acid substitution in the NSs at position 102 (P102A) [47]. The proline at position 102 is located in an amino acid sequence motif (S_97_xLRWPxG_104_) that is conserved in the NSs coding region within pathogenic phleboviruses. These mutant viruses lost the ability to induce tumor progression locus 2 (TPL2) signaling and interleukin (IL)-10 production and resulted in a highly attenuated phenotype in mice. These two recombinant viruses showed an attenuated phenotype and induced a humoral immune response in ferrets. After vaccination with attenuated SFTSVs, ferrets were also completely protected against a lethal challenge (10^7.6^ TCID_50_) with SFTSV strain CB1/2014, which is a different genotype of HB29.

### 3.2. Recombinant Viral Vector-Based Vaccines

Viral vector vaccines are based on recombinant viruses. Viruses for the vectors are low-pathogenic viruses or vaccine strains for other viral infectious diseases. These recombinant viruses are sometimes further attenuated to reduce pathogenicity and retain immunogenicity.

#### 3.2.1. rVSV-Based Vaccines

Indiana vesiculovirus, formerly vesicular stomatitis virus (VSV) is a zoonotic arbovirus that belongs to the family Rhabdoviridae. Cattle, horses, and swine infected with VSV develop severe disease, and swine show symptoms that are similar to those of foot and mouth disease, swine vesicular disease, and vesicular exanthema of swine. There are few reports of human VSV infections. Thus, most infections in humans are thought to be asymptomatic. Even if it is symptomatic, the symptoms are generally not severe, although there is a report of a child who developed encephalitis [48]. Thus, VSV is a potent candidate for recombinant vaccine vectors. Recombinant VSV (rVSV) expressing Ebola virus glycoprotein was evaluated in clinical trials and its efficacy in the prevention of Ebola viral disease confirmed [49]. The rVSV-based vector induces strong humoral [50] and potent cellular immune responses against pathogens [51]. The vector does not induce vector-specific humoral immunity and therefore does not lose efficacy upon repeated application [52].

Dong et al. reported the efficacy of a live attenuated rVSV expressing the SFTSV Gn/Gc glycoproteins (rVSV-SFTSV) as a vaccine candidate for SFTS [53]. In rVSV-SFTSV, the authentic envelope glycoprotein (VSV-G) gene was substituted for the human codon-optimized glycoprotein Gn/Gc open reading frame of the Chinese clade of SFTSV. Thus, the virus expresses the SFTSV Gn/Gc glycoproteins on the virion surface and uses it as the viral receptor-binding protein instead of the VSV-G. Mice that were intraperitoneally vaccinated with rVSV-SFTSV elicited neutralizing antibodies to SFTSV, and interestingly, the antibodies also neutralized the Heartland virus, which is closely related but distinct from SFTSV [54]. *Ifnar^−^*^/*−*^ mice that were vaccinated with a single dose of 2 × 10^4^ PFU rVSV-SFTSV were also completely protected from an SFTSV challenge with 2 × 10^4^ focus forming units (FFU), which was a lethal dose to naïve and rVSV expressing Hantaan virus glycoprotein (rVSV-HTNV)-vaccinated mice. rVSV-SFTSV also completely protected aged *Ifnar^−^*^/*−*^ mice (age: 8 to 9 months) against a lethal SFTSV challenge, and the result suggested that vaccination with rVSV-SFTSV would provide effective protection from SFTS in elderly people. In naïve mice, passive transfer of 200 µL of sera from 2 × 10^4^ PFU of rVSV-SFTSV-immunized mice conferred protection against a challenge with 2 × 10^3^ FFU of SFTSV. The result demonstrated that rVSV-SFTSV elicited strong humoral immunity in mice. rVSV expressing Heartland virus Gn/Gc also conferred complete cross-protective immunity against a lethal challenge with 2 × 10^4^ FFU of SFTSV, as was expected based on the induction cross-reactive neutralizing antibodies. Incidentally, since rVSV has been used as a vaccine vector for several infectious diseases—as mentioned above—it is possible that an individual may receive rVSV-based vaccines for several infectious diseases. Thus, the authors evaluated the effect of pre-existing anti-VSV immunity on the efficacy of rVSV-SFTSV. *Ifnar^−^*^/*−*^ mice were vaccinated with 2 × 10^4^ PFU of rVSV-HTNV. Thirty days later, the mice were vaccinated with 2 × 10^4^ PFU of rVSV-SFTSV. After another 30 days, the mice were challenged with 2 × 10^4^ FFU of SFTSV. The mice pre-immunized with rVSV-HTNV showed approximately 10% weight loss and recovered within 7 days after the SFTSV challenge, while the untreated, control animals died on post-challenge days 3–4. The result indicated that the efficacy of rVSV-SFTSV was not affected by pre-existing VSV-specific immunity.

#### 3.2.2. rVAC-Based Vaccines

VAC was previously used as a vaccine for smallpox and has been used as a recombinant vaccine vector with the expectation of immunogenicity [55,56]. Unfortunately, after these strains, the so-called second generation of VAC used during the eradication campaign was associated with severe adverse events [55,57,58], the third generation of VAC strains, which included LC16m8 (m8) and modified vaccinia Ankara (MVA), was established. m8 is confirmed to be highly attenuated while still maintaining immunogenicity as well as MVA [59,60,61] and is licensed for use in healthy people in Japan. At present, approximately 100,000 people have undergone vaccination with m8 without experiencing any severe postvaccine complications [62,63]. The significant difference in the characteristics between m8 and MVA is the replication capacity. m8 can infect mammalian cells and produce progeny viruses in rabbit kidney-based cells, such as primary rabbit kidney cells and RK13 cells, although the host range of cell types is restricted. On the other hand, MVA can infect mammalian cells but cannot replicate well in most mammalian cells [58]. At present, the third generation of VAC strains are attractive as recombinant vaccine vectors, especially for viral hemorrhagic infectious diseases, such as Ebola virus disease, Lassa fever, Crimean-Congo hemorrhagic fever, and SFTS.

A study reported the possibility of recombinant m8 strains that possess the coding regions of SFTSV N (m8-N), GPC (m8-GPC), or both N and GPC (m8-N+GPC) (m8-based SFTSV vaccines) as SFTS vaccine candidates [64]. These m8-based SFTSV vaccines express SFTSV genes in the infected cells, and especially the cells infected with m8-GPC or m8-N+GPC produce SFTS VLP in culture supernatant in vitro. Specific antibodies to SFTSV were induced in *Ifnar^−^*^/*−*^ mice when they were subcutaneously vaccinated twice with m8-based SFTSV vaccines (1 × 10^6^ PFU each time). Furthermore, *Ifnar^−^*^/*−*^ mice that were vaccinated twice with the m8-based SFTSV vaccine (1 × 10^6^ PFU each time) were fully protected from challenges with either 10^3^ TCID_50_ or 10^5^ TCID_50_ of SFTSV, which represented lethal doses to naïve mice and recombinant m8 possessing the coding region of enhanced green fluorescent protein (m8-EGFP)-vaccinated mice. The serum infectious virus titer and viral copies in the mice vaccinated with m8-N, m8-GPC and m8-N+GPC were drastically decreased in comparison to those of m8-EGFP-vaccinated mice. Incidentally, in many countries, routine smallpox vaccination ended between the early 1970s and 1980 [65]. In other words, people born before 1980 possibly have immunity against smallpox virus infection and VAC infection. Hence the impact of pre-existing immunity to VAC on the protective immunity induced by m8-based SFTSV vaccines was evaluated. *Ifnar^−^*^/*−*^ mice were immunized with VAC strain Lister, a strain widely used during a global smallpox eradication campaign and were then vaccinated with each of the m8-based SFTSV vaccines and challenged with SFTSV. The survival rate of the VAC-preimmunized mice infected with either 1 × 10^3^ or 1 × 10^5^ TCID_50_ of SFTSV was significantly improved by vaccination with m8-N, m8-GPC, or m8-N+GPC in comparison to the control mice vaccinated with m8-EGFP, although the rates were lower than those of mice that were not immunized with VAC Lister in advance of the vaccinations. The survival rates of m8-GPC- or m8-N+GPC-vaccinated mice were also higher in comparison to m8-N-vaccinated mice, and thus the results suggested that GPC was better than N as the antigen for SFTS vaccine. To verify the contribution of the humoral immunity induced by the m8-SFTSV vaccines, 400 µL of pooled sera obtained from the mice vaccinated twice with 1 × 10^6^ PFU of m8-based SFTSV vaccines was intraperitoneally administered to naïve mice on −1, 0, and 1 days post-infection with SFTSV. Although the body weight change seemed to be improved in the m8-N-, m8-GPC- and m8-N+GPC-vaccinated mouse sera-treated mice on day 5 post-infection, a statistically significant improvement in comparison to the control (m8-EGFP-mouse sera-treated mice) was only achieved in the m8-N+GPC-mouse sera-treated mice. These results suggested that the transfer of both the humoral immunity against SFTSV GPs and N simultaneously contributed—to a certain degree—to conferring anti-SFTSV protective immunity, although the contribution of cellular immunity remains uncertain.

### 3.3. Protein Subunit Vaccines

There are few reports evaluating the efficacy of whole-inactivated SFTSV or SFTSV protein subunits as vaccine candidates. Since the humoral immunity induced by some of the live vaccines as mentioned above was effective, a certain degree of efficacy is expected if a whole-inactivated SFTSV, SFTS VLP, or SFTSV GPs subunit is used as a vaccine component. Nevertheless, Liu et al. evaluated the efficacy of SFTSV nonstructural protein (NSs) as a vaccine component [66]. Immunocompetent C57BL/6 mice were immunized with 100 μg of purified recombinant NSs expressed by *E. coli* with Freund’s complete adjuvant and then challenged with SFTSV. The viremia level in the NSs vaccinated mice was not significantly different from the control mice. Hence the authors concluded that vaccination with NSs did not promote clearance of SFTSV in mice.

### 3.4. DNA-Based Vaccines

DNA vaccines usually consist of a plasmid DNA encoding the vaccine antigen driven by the mammalian promoter. For the efficient uptake of the plasmid DNA into cells to occur, the inoculation needs to follow an electroporation procedure in vivo. The protein expression mechanism from the plasmid DNA was similar to that of viral proteins during viral infection. Hence, a DNA vaccine can induce both humoral and cellular immunity for recognition by B cells and presentation by major histocompatibility complex (MHC) class I and II molecules.

Kwak et al. reported the efficacy of a DNA vaccine in a ferret model of SFTS [67]. DNA plasmids encoding the full-length Gn, Gc, N, NSs, and RdRp genes of SFTSV, named pVax1-Gn, pVax1-Gc, pVax1-N, pVax1-NSs, and pVax1-RdRp, were generated. The genes encoded in the plasmid were driven by human cytomegalovirus (CMV) immediate early enhancer and promoter. To evaluate the vaccine efficacy, aged-ferrets (age: > 4 years) were vaccinated with a mixture of all five SFTSV DNA vaccines (pVax1-Gn, pVax1-Gc, pVax1-N, pVax1-NSs, and pVax1-RdRp) three times at two-week intervals via intradermal injection, which was followed by in vivo electroporation at the site of delivery. Two weeks after the last vaccination, the ferrets were intramuscularly challenged with 10^7.6^ TCID_50_ of SFTSV. In comparison to the control group, which was vaccinated with the control plasmid backbone (modified pVax1), ferrets vaccinated with the SFTSV DNA vaccines were fully protected from the challenge. The serum SFTS viral load in ferrets vaccinated with the SFTSV DNA vaccines was below the limit of detection during eight days after the SFTSV challenge, whereas the peak viral load in the ferrets vaccinated with the control plasmid reached 10^4^ copies/mL serum on day four post-infection. Further studies revealed that a mixture of envelope glycoproteins (pVax1-Gn and pVax1-Gc) or non-enveloped proteins (pVax1-N, pVax1-NSs, and pVax1-RdRp) was sufficient to confer complete protection to the ferrets against a lethal infection of SFTSV. In naïve ferrets, passive transfer of sera from ferrets vaccinated with pVax1-Gn and pVax1-Gc—but not from mice vaccinated with pVax1-N, pVax1-NSs, pVax1-RdRp—could confer effective protection against lethal infection. Interestingly, monovalent vaccination of either pVax1-N, pVax1-NSs, or pVax1-RdRp drastically reduced the vaccine efficacy; this result indicates that three or—at least—two of these non-enveloped proteins are necessary to confer complete protection.

The other DNA vaccine candidate was reported by Kang et al. [68]. This DNA vaccine increased the protection efficacy via the simultaneous expression of interleukin (IL)-12, an essential cytokine for the induction of T-helper 1 (Th1) and cellular immunity. The plasmid, pSFTSV, encodes ectodomains of Gn, Gc, and NP/NSs fusion protein genes driven by Rous sarcoma virus (RSV) and CMV promoters, respectively, and pSFTSV-IL-12, which was based on pSFTSV, additionally encodes the IL-12 alpha and beta gene driven by the pEF1alpha promoter. *Ifnar^−^*^/*−*^ mice were vaccinated with either mock vector, pSFTSV, or pSFTSV-IL12 three times via intramuscular injection, followed by in vivo electroporation at the site of delivery, and then subcutaneously challenged with a lethal dose (10^5^ FFU) of SFTSV. Mice vaccinated with pSFTSV-IL12 or pSFTSV were fully or partially protected, respectively, from a challenge with a lethal dose of SFTSV, whereas the mock-vaccinated mice succumbed within five days post-infection. This result indicated that the expression of IL-12 improved the efficacy of the DNA vaccine. Consistently, mice vaccinated with pSFTSV-IL12 lost weight until day four post-infection and then recovered, whereas the bodyweight of pSFTSV-vaccinated mice decreased until day eight post-infection, and then recovered in the surviving mice. On day four post-infection, the blood viral load of mice vaccinated with pSFTSV-IL12 and pSFTSV was significantly lower in comparison to mock-vaccinated mice.

## 4. Conclusions

Taken together, the efficacy of several promising SFTS vaccine candidates has been confirmed in small animal models. A better nonhuman primate model of SFTS is needed. The cat model is available to evaluate the efficacy of these candidate SFTS vaccines on a domesticated animal. Indeed, these SFTS vaccines will be put to use in the foreseeable future.
